# Poly(Acrylic Acid)/TiO_2_ Nanocomposite Hydrogels for Paper Artwork Cleaning and Protection

**DOI:** 10.3390/molecules30010075

**Published:** 2024-12-28

**Authors:** Sabina Botti, Francesca Bonfigli, Rosaria D’Amato, Jasmine Rodesi, Maria Gabriella Santonicola

**Affiliations:** 1 ENEA C.R. Frascati, Photonics Micro- and Nano-Structures Laboratory, Physical Technologies and Security Division, Nuclear Department, Via E. Fermi 45, 00044 Frascati, Italy; francesca.bonfigli@enea.it (F.B.); rosaria.damato@enea.it (R.D.); 2 Department of Chemical Engineering Materials Environment, Sapienza University of Rome, Via del Castro Laurenziano 7, 00161 Rome, Italy; rodesi.jasmine@gmail.com (J.R.); mariagabriella.santonicola@uniroma1.it (M.G.S.)

**Keywords:** Raman spectroscopy, cultural heritage, paper aging diagnostics, Raman spectral imaging, hydrogel paper cleaning

## Abstract

Paper-based artworks are prone to natural aging processes driven by chemical and biological processes. Numerous treatments have been developed to mitigate deterioration and prevent irreversible damage. In this study, we investigated the use of poly(acrylic acid)/TiO_2_ composite hydrogels, combining their cleaning and protective functions in a minimally invasive treatment. Hydrogels allow for controlled water flow and photocatalytic TiO_2_ nanoparticles enhance the hydrogel’s efficacy by enabling the removal of oxidation products and inactivating biological contaminants. Furthermore, this innovative material can act as a protective coating against UV-induced aging, preserving both color and stability of the paper. Raman spectroscopy and confocal laser scanning microscopy imaging techniques were employed to evaluate the treatments, allowing for us to differentiate between hydrolytic and oxidative aging processes. Our findings demonstrate that papers coated with poly(acrylic acid)/TiO_2_ composite hydrogels exhibit significant reductions in oxidative markers, an enhanced color stability, and an overall improved resistance to degradation compared to uncoated samples.

## 1. Introduction

Nowadays, physical and chemical technologies are applied in the study, conservation, and restoration of ancient artifacts. It is of paramount importance to acquire expertise in performing restoration treatments that slow down the aging effects of cultural artifacts preserving the artifact’s unique characteristics. In this regard, the development of non-destructive techniques to assess an artifact’s condition before and after treatment is a crucial step to achieve this goal. Spectroscopic techniques have played a pivotal role, particularly in the field of book heritage [1,2,3,4,5,6,7,8,9].

The natural degradation process of paper over time results in both chemical and physical alterations. The primary constituent of paper, cellulose, undergoes two main degradation mechanisms through hydrolytic and oxidative reactions [10]. Hydrolysis can occur in both acidic and basic environments causing depolymerization, whereas oxidation causes functional groups to bind to the cellulose, also disrupting its structure. The rate at which these processes occur is affected by several factors:

- Intrinsic characteristics of the paper, including the raw materials used during production and manufacturing methods.
- Nature of materials applied to the paper such as inks, pigments, binders, etc.
- Possible presence of pathogens and air pollutants in the storage environment, as well as light exposure.

To preserve valuable artwork, it is essential to slow down and prevent degradation phenomena by implementing controlled restoration and protection techniques. Among the various paper restoration operations, cleaning plays a fundamental role. The aim of cleaning is to remove all cellulose degradation products as well as contaminant particles from the paper surface—in other words, materials that can alter the paper appearance and cause further aging.

Cleaning treatments involving immersion in water are widely employed, as water effectively solubilizes most of the foreign particles on the surface, penetrating deeply in between cellulose fibers. Moreover, water promotes the formation of hydrogen bonds between cellulose fibrils, improving the bending resistance of paper sheets. However, washing treatments bring up some concerns: the uncontrolled water flow may meet more degradable constituents or trigger unwanted processes, potentially causing irreversible alterations to the appearance of the artifact and the historical information it conveys.

Lately, materials of a certain texture such as hydrogels have been used to control volume and speed of water flow. Hydrogels are not reactive and retain large volumes of water and/or other solvents, releasing them on the paper surface upon contact. These solvents dissolve the target substances, which are subsequently re-adsorbed by the hydrogels together with the solvents used.

The use of water alone in hydrogels has proven highly effective to clean water-soluble materials commonly found in book artifacts, such as polysaccharides (e.g., starch, gum arabic) and protein-based substances (e.g., gelatin, animal glues). For the removal of non-water-soluble substances, alternative solvents can be employed. Nevertheless, hydrogels make localized treatments on specific areas possible, significantly reducing exposure of operators to potentially harmful vapors. The use of hydrogels as supporting materials is well documented in the literature [11,12].

In this study, we employed a poly(acrylic acid) (PAAc) hydrogel, commercially available under the trade name Carbopol (CBP), loaded with TiO_2_ nanoparticles for restoration purposes. Derived from polyacrylic acid, CBP can form gels with good viscosity already at low concentrations (1–1.5% *w*/*v*) [13,14], which guarantees reduced adhesive capacity and, hence, easy removal. The incorporation of TiO_2_ nanoparticles enhances the hydrogel’s cleaning efficacy due to their photocatalytic properties. TiO_2_ nanoparticles enable the inactivation of bacteria, fungi, and algae, as well as the removal of organic contaminants [12,14,15,16,17]. Moreover, TiO_2_ is considered safe and harmless to humans.

The synthesis of TiO_2_ thin coatings typically employs high temperatures or mechanical conditions incompatible with paper substrates [18]. In a previous study, TiO_2_–cellulose nanocomposite was deposited via spray techniques and was employed as paper consolidant to inhibit aging effects induced by Ultra Violet (UV) light, air pollutants, and microbial activity [19].

In this work, we incorporated laser synthesized TiO_2_ nanoparticles with high chemical purity into a PAAc (CBP) matrix to create a composite hydrogel that could be applied on various paper samples [13,20] using a spatula. Our aim was to investigate the dual functionality of composite gel as cleaning agent and protective coating of paper artwork. Two experimental approaches were then followed:

In the first, the CBP/TiO_2_ hydrogel was used as a cleaning agent. After application, the composite gel was activated by UV irradiation at 385 nm. When in contact with the porous surface of paper, the gel gradually releases water, facilitating cleaning. We conducted experiments with varying contact times to determine the optimal cleaning conditions that ensure removal of oxidation products and contaminants without compromising the structural integrity of the paper. After removal of the gel, residues can be found on the treated surfaces. Their presence was assessed via confocal Raman spectroscopy with surface scanning.

In a second experimental procedure, a thin layer of CBP/TiO_2_ gel was applied to the paper without removal after UV activation. This approach aimed to evaluate its performance as a protective coating against light-induced aging.

The effects of the cleaning/protective procedures were analyzed by confocal Raman spectroscopy with surface scanning and confocal laser scanning microscopy (CLSM). In a previous study [7], we identified the following paper aging markers based on Raman measurements:

*R*_H_ = I_1100cm_^−1^/I_1380cm_^−1^ (1)

*C*_I_ = I_2890cm_^−1^/I_1380cm_^−1^ (2)

*O*_I_ = A_1640–1850cm_^−1^/A_1500–1600cm_^−1^ (3)

*O*_T_ = A_1500–2800cm_^−1^/A_700–3000 cm_^−1^ (4)

where I and A indicate intensity and area of peaks in the selected Raman shift range, respectively.

These markers account separately for the hydrolysis and oxidation processes on cellulose, the main constituent of paper structure. Hydrolysis, which shortens cellulose chains, leads to a decrease of the *R*_H_ marker, proportional to the degree of polymerization, and of the crystallinity index *C*_I_ [7]. Oxidation processes, which involve the addition of functional groups to the cellulose backbone, lead to an increase of the *O*_T_ marker, followed by an increase of the *O*_I_ marker, which is directly proportional to the area of carbonyl bands (A_1640–1850_), representing the final oxidation stages of cellulose and, therefore, giving insight on the advancement state of the whole oxidation process.

Their variation in percentage was used to evaluate the cleaning/protective procedure efficiency. Comparisons between treatments involving the CBP/TiO_2_ composite gel activated with UV irradiation and the unmodified CBP gel showed a significant reduction in oxidation markers, whereas polymerization degree and crystallinity markers remained unaffected, showing that paper integrity was preserved.

Additionally, our measurements demonstrated that the CBP/TiO_2_ coating effectively counter acted oxidation, slowed down hydrolysis maintaining color stability, and avoiding aging yellowing.

## 2. Results

### 2.1. Characterization of Cleaning Process with Raman Micro-Spectroscopy and Confocal Laser Microscopy

**Figure 1:**
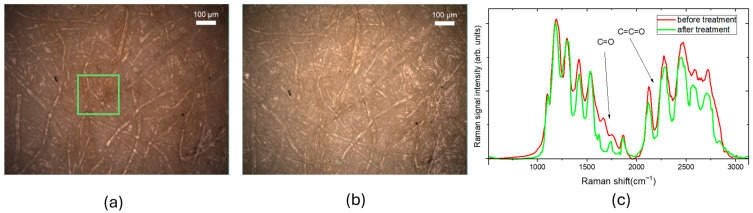
Optical images in bright field of XIX paper. The green rectangle indicates the area of 200 µm × 200 µm scanned with a step size of 5 µm in Raman spectral imaging mode using green excitation (λ = 532 nm) and 10X objective (**a**) before the cleaning treatment; (**b**) after the cleaning treatment; (**c**) examples Raman spectra acquired before and after the cleaning treatment.

In our experiments, we analyzed different paper samples. Figure 1 shows white light optical images of a 19th-century paper sample before (Figure 1a) and after (Figure 1b) the cleaning treatment. The green square marks the 200 µm × 200 µm area scanned via Raman spectral imaging mode. Figure 1c shows the typical Raman spectra detected in a point of the square.

The main peaks observed in the Raman spectra are attributed to vibrational modes of carbon-based species. These include C–C stretching and C–O–H and C–C–H vibrations in the 1000–1350 cm^−1^ range, as well as lower intensity in-plane bending vibrations of H–C–H and O–C–H at 1425 cm^−1^ [7,9,21,22]. The peaks with wavenumber greater than 1500 cm^−1^ are due to formation of oxidized molecular groups (excluding CH-CH_2_ stretching peaks at 2850–3000 cm^−1^). In particular, the bands at 1640 and 1740 cm^−1^ are, respectively, ascribed to C=O stretching in carbonyl and carboxyl groups. The band at 1550 cm^−1^ is ascribed to symmetric C=C stretching in C=C–O structures, while the peak at 1850 cm^−1^ represents an overlap between C=O stretching and allene C=C=C stretching. Vibrations in the 2000–2100 cm^−1^ region are attributed to C=C=O stretching of ketene groups. Finally, the peaks around 2550 cm^−1^ are associated with overtones and combinations of carboxylic group frequencies.

As shown in Figure 1c, following the cleaning treatment, the intensity of C=C-O and C=O group peaks decreases indicating that the gel application removes the oxidation products from the paper surface.

The cleaning treatments were carried out by applying the hydrogel onto the paper samples, with contact times of up to 1 h. Some additional tests were performed with a contact time of 2 h, but the changes in the markers were not significantly different from those observed after 1 h and transferring and excessive amount of water onto the paper surface was also a risk. During the cleaning treatment involving the CBP/TiO_2_ nanoparticles, the gel was irradiated with UV light for the duration of the treatment.

For each spectrum in the Raman map, both before and after gel application, the values of the markers defined in (1)–(4) were calculated, obtaining a distribution of marker values for each map.

**Figure 2:**
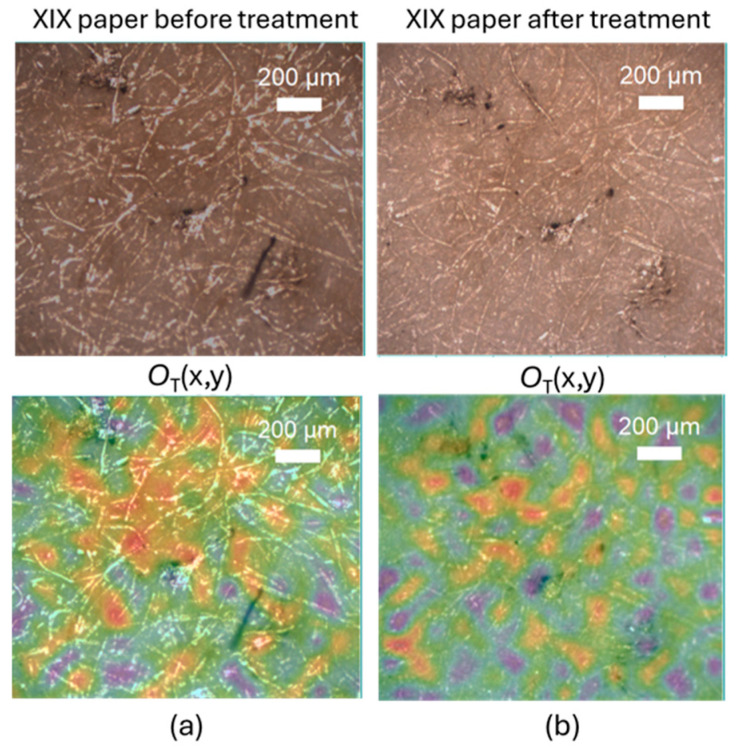
Optical images of XIX paper (**upper** panels) and *O*_T_ marker value colocalized maps. (**a**) Before and (**b**) after 1 h CBP cleaning treatment. The color code is blue, green, red, yellow for increasing value of *O*_T_ marker.

As an example, Figure 2 shows the *O*_T_ marker maps, in false colors from lower values in green to higher values in yellow-red, co-localized with the optical image of the XIX century paper sample: a large dark stain is visible in Figure 2a, which becomes lighter after cleaning, as shown in Figure 2b. Contact time was 1 h. By monitoring the peaks associated with the formation of oxidized molecular groups using the *O*_T_ marker, we can clarify the activity of CBP. Following the cleaning treatment, the yellow-red areas of the paper, corresponding to higher *O*_T_ values, decreased.

**Figure 3:**
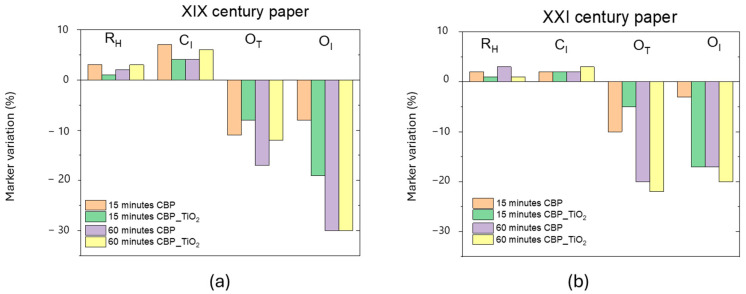
(**a**) Ageing marker variation in percentage for the XIX century paper, (**b**) the same for the XXI century paper. This laser printer 2021 paper was exposed to ambient light for three years.

For each marker map, we calculated the average marker values before and after the CBP cleaning treatments, with and without the addition of TiO_2_ nanoparticles. Figure 3 illustrates the observed changes in the marker values for the two paper samples used in our study: a XIX century paper sample as well as a 2021 laser printer paper that had been exposed to ambient laboratory light for three years.

Although the most pronounced effects on marker values were observed in the XIX century paper, both treatments resulted in an increase in the *C*_I_ and *R*_H_ markers. This finding can be attributed to the formation of hydrogen bonds between cellulose fibers facilitated by the hydrogel’s water content. It was shown that after washing and drying processes the formation of additional hydrogen bonds can occur, enhancing some mechanical properties of the paper, such as bending resistance.

Oxidation markers decreased significantly (−20% *O*_T_ and −30% *O*_I_ after 1 h of treatment with CBP with and without TiO_2_ addition). The reduction in *O*_I_, which is proportional to the carbonyl content, suggests that oxidation products containing C=O groups were primarily removed. This is particularly noteworthy because such carbonyl-containing groups contribute to the formation of chromophore molecules responsible for the yellowish color typical of aged paper.

Although the application of the CBP/TiO_2_ hydrogel leads to a noticeable decoloration of the paper (see Figure 1 and Figure 2), there is no great improvement in the decrease of oxidation markers with the use of the composite hydrogel instead of the CBP. In this respect, the advantage to use the composite gel is that the presence of TiO_2_ nanoparticles provides a biocidal effect.

**Figure 4:**
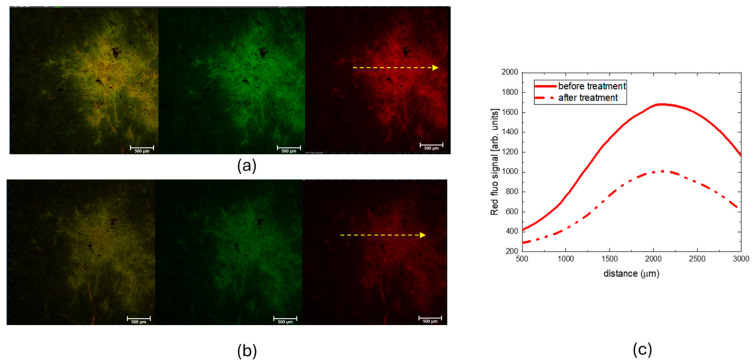
CLSM images (objective 4×) of a selected spot on Brehm paper. (**a**) Before cleaning treatment; (**b**) after cleaning treatment with CBP/TiO_2_. The figures show red and the green fluorescence channels and their overlay in yellow color (**c**) Graph of the red fluorescence signal intensity profiles detected along the yellow arrows of the spot before and after CBP/TiO_2_ treatment.

Figure 4 shows the CLSM images obtained in fluorescence mode, showing both red and green fluorescence channels, as well as their merge, from a selected region of the 19th-century paper sample before (Figure 4a) and after (Figure 4b) the CBP/TiO_2_ cleaning treatment. The graph on the right displays the intensity profiles of the red fluorescence signal, measured along the yellow arrows.

It is known that one of the effects of paper aging is the formation of products like simple sugars, cellulose oligomers, and phenolic compounds originating from the degradation of cellulose, hemicellulose, and lignin [23,24]. These species have a strong luminescence with a broad band; therefore, the observed decrease in fluorescence intensity, as evidenced by the CLSM measurements, indicates that, besides oxidized groups, the CBP/TiO_2_ treatment eliminates from the paper surface also the cellulose degradation products.

**Figure 5:**
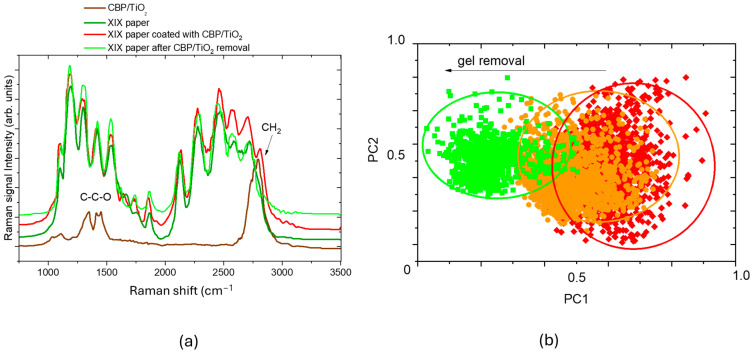
(**a**) Raman spectra of CBP/TiO_2_ coated XIX century paper (red curve), XIX century paper sample after gel removal (light green curve), XIX century paper (green curve), and CBP/TiO_2_ gel (brown curve); (**b**) score plot related to map of CBP/TiO_2_ coated XIX century paper (red diamonds); CBP/TiO_2_ coated XIX century paper after a first run of gel removal (orange dots) and a second run of gel removal (green squares). Ellipses are only a guide for the eyes.

Figure 5a reports the comparison between the Raman spectra of CBP/TiO_2_ coated XIX century paper (red curve), uncoated paper (green curve), and CBP/TiO_2_ gel (brown curve). As evidenced by the appearance of a new peak around 2800 cm^−1^, ascribed to CH_2_ vibration in CBP, the Raman spectrum measured from the coated paper sample can be expressed as a linear combination (classical least square fitting, CLS) of the CBP/TiO_2_ gel spectrum (PC1 component) and the uncoated paper (PC2 component). The coefficients (scores) of the linear combination are reported in a x,y plane in Figure 5b as red diamonds.

When we progressively removed the gel by spatula, we observed a decrease in CH_2_ peak intensity and a concomitant decrease of PC1 component, allowing for us to monitor and evaluate the effectiveness of gel removal and determine whether it was sufficient or not.

The gel left on the paper could prolong the protective activity against aging agents. In this regard, additional tests were performed by applying a thin layer of the CBP/TiO_2_ gel onto the paper, aiming to evaluate its performance as protective layer, as discussed in the following paragraph.

### 2.2. Characterization of Protective Effect of CBP/TiO_2_ Coating

**Figure 6:**
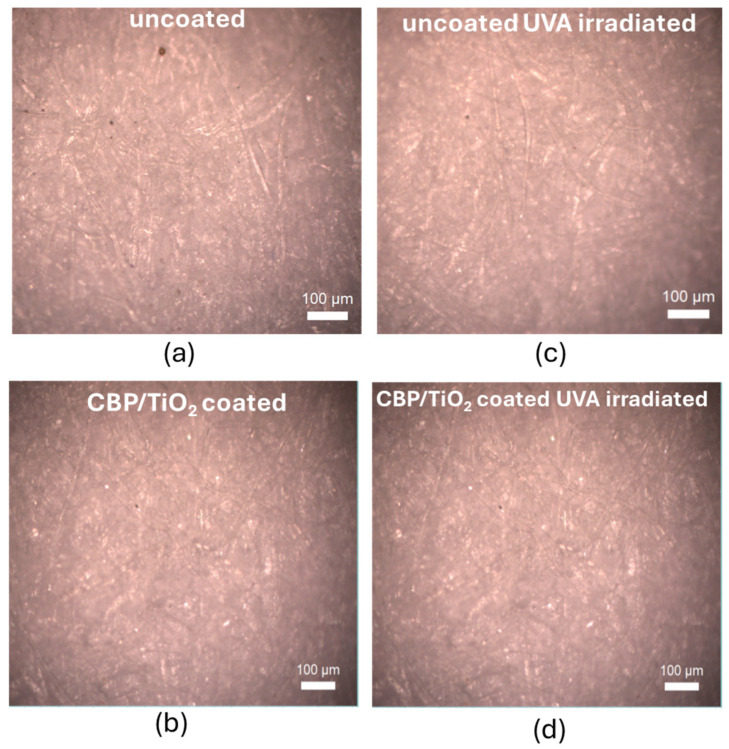
Optical images of 2024 modern paper: (**a**) uncoated and non-irradiated; (**b**) coated with a thin layer of CBP/TiO_2_; (**c**) uncoated and UVA irradiated; (**d**) coated with a thin layer of CBP/TiO_2_ and UVA irradiated.

To evaluate the suitability of the CBP/TiO_2_ coating as a protective layer for paper, we used two samples of modern (2024) laser printer paper. One sample was coated with a thin layer of CBP/TiO_2_ gel applied with a spatula, while the other remained uncoated. Both samples were aged by ultraviolet A irradiation at 385 nm (UVA) for 31 h. The optical images, shown in Figure 6, were subsequently analyzed using ImageJ software to evaluate RGB value distributions of the images.

**Figure 7:**
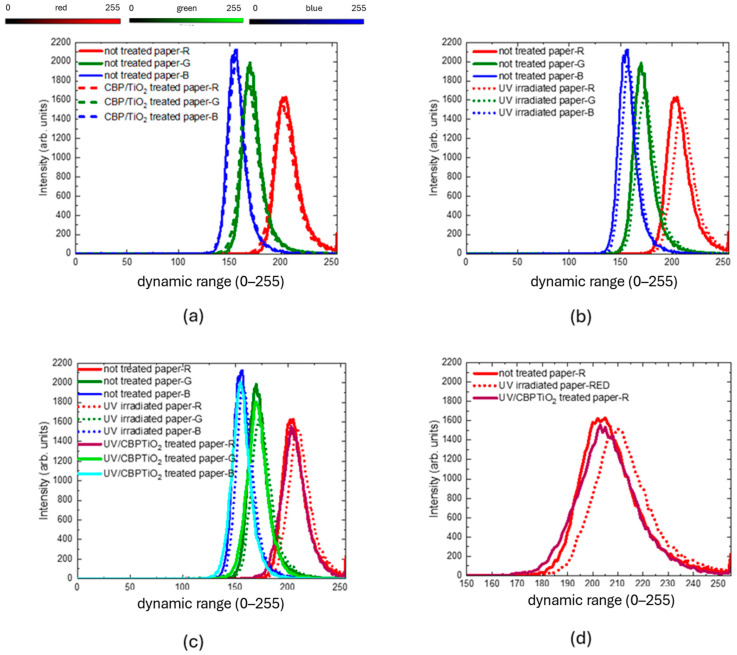
Red–green–blue value distributions of 2024 modern paper comparing (**a**) uncoated (solid lines) and CBP/TiO_2_ coated paper (dashed line); (**b**) uncoated paper (solid lines) and uncoated paper UVA-irradiated (dotted lines); (**c**) uncoated paper (solid lines), uncoated UVA-irradiated paper (dotted lines), CBP/TiO_2_ coated UVA-irradiated paper (brown, light green, and cyan solid lines). (**d**) Magnification of red value distribution of uncoated paper (solid line), uncoated UVA-irradiated paper (dotted line), uncoated UVA irradiated paper (brown solid line).

The RGB histogram of the coated and uncoated samples, shown in Figure 7a, indicates that the CBP/TiO_2_ coating is a transparent layer that does not significantly alter the color of the paper.

As shown in Figure 7b, prolonged exposure to UVA irradiation leads to the yellowing of uncoated paper, as demonstrated by a shift towards higher RGB values, in particular of the red component. In contrast, RGB values of the coated paper after UVA irradiation (see Figure 7c,d) remain similar, showing that the CBP/TiO_2_ coating maintains the paper color. This property can be exploited for the protection of the art works against light.

**Figure 8:**
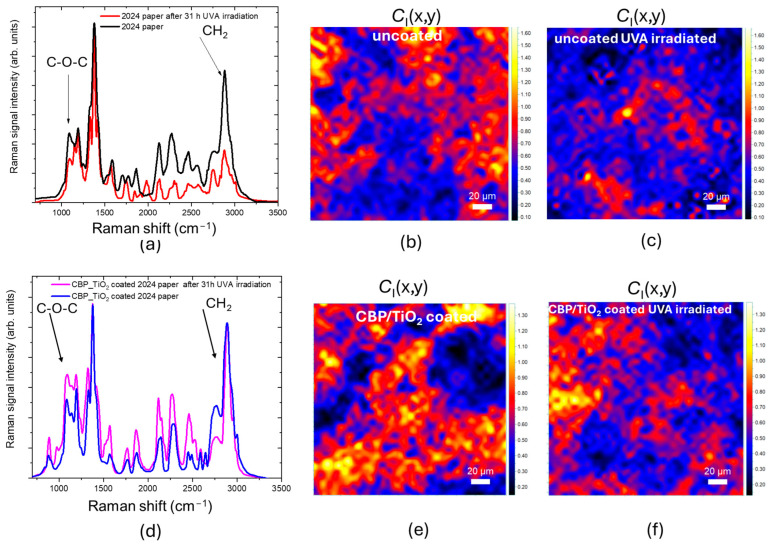
(**a**) Raman spectra acquired from 2024 paper before and after 31 h of UVA irradiation. (**b**,**c**) *C*_I_ marker value maps of uncoated 2024 paper non-irradiated and UVA-irradiated, respectively. (**d**) Raman spectra acquired from 2024 paper CBP/TiO_2_ coated before and after 31 h of UVA irradiation. (**e**,**f**) *C*_I_ marker value maps of CBP/TiO_2_ coated 2024 paper, non-irradiated and UVA-irradiated, respectively.

As shown in Figure 8a, the Raman spectra acquired from the 2024 paper before and after 31 h of UVA irradiation exhibit a significant difference, particularly in the C–O–C and CH_2_ peaks, which are notably reduced after UVA exposure. The C-O-C peak related to the stretching of bond between monomers of cellulose, is proportional to the cellulose chain length (*R*_H_ marker) whereas the CH_2_ peak is related to the order of fibrils (crystallinity degree, *C*_I_ marker). The decrease in these peaks, and hence of corresponding markers, indicates that the uncoated paper has undergone a light-induced degradation of cellulose via hydrolysis. The shortening of the cellulose chains is accompanied by an increase in disorder, measured by the decrease in the *C*_I_ marker, shown in Figure 8b.

Differently, for the 2024 coated paper, the decrease in the C–O–C and CH_2_ peaks is less pronounced (Figure 8d–f).

**Figure 9:**
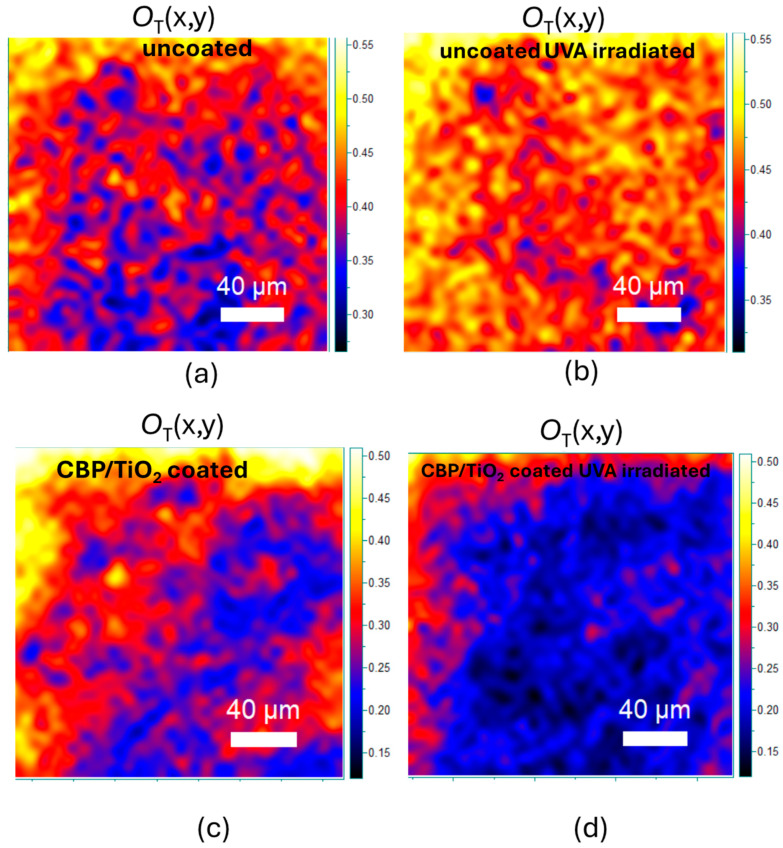
(**a**,**b**) O_T_ marker value maps of uncoated 2024 paper before and after 31 h of UVA irradiation, respectively. (**c**,**d**) O_T_ marker value maps of CBP/TiO_2_ coated 2024 paper before and after 31 h of UVA irradiation, respectively.

The protective effect of the coating against cellulose oxidation was also assessed. As shown in Figure 9a,b, after UVA exposure, the O_T_ marker increases, due to the incorporation of oxidized functional groups into the cellulose backbone.

As for the coated paper, the O_T_ marker decreases (Figure 9c,d), suggesting that the UVA irradiation may prolong the cleaning action of the gel.

**Figure 10:**
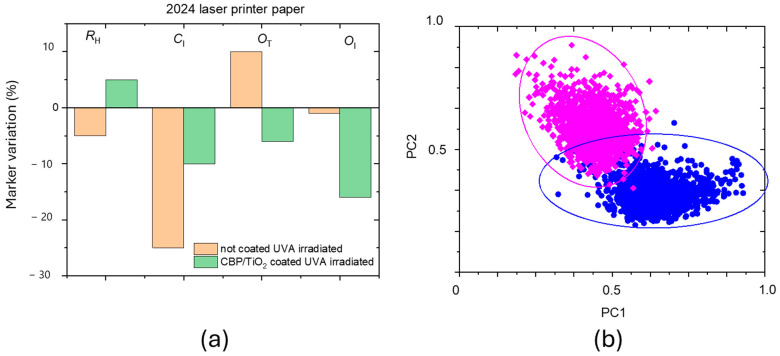
(**a**) Marker variation in percentage after 31 h of UVA irradiation for the uncoated and coated 2024 paper. (**b**) Score plots of coated paper before (blue points) and after irradiation (fuchsia points). The PC1 component is the CBP/TiO_2_ gel Raman spectrum, while PC2 component is the paper one.

The marker variation before and after 31 h in UVA irradiation is shown in percentage in Figure 10 for both uncoated and coated 2024 paper samples. The CBP/TiO_2_ coating not only inhibits the oxidation and hydrolysis of cellulose but also inhibits the decrease of *C*_I_.

Figure 10b illustrates the score plots for coated samples before (blue dots) and after (fuchsia dots) UVA irradiation. The observed reduction in gel scores may be attributed to the gel depletion on the paper surface caused by UVA exposure. However, as observed in previous experiments [13], an alternative hypothesis to consider is the penetration of the gel layer beneath the outer fiber of the sample surface.

**Figure 11:**
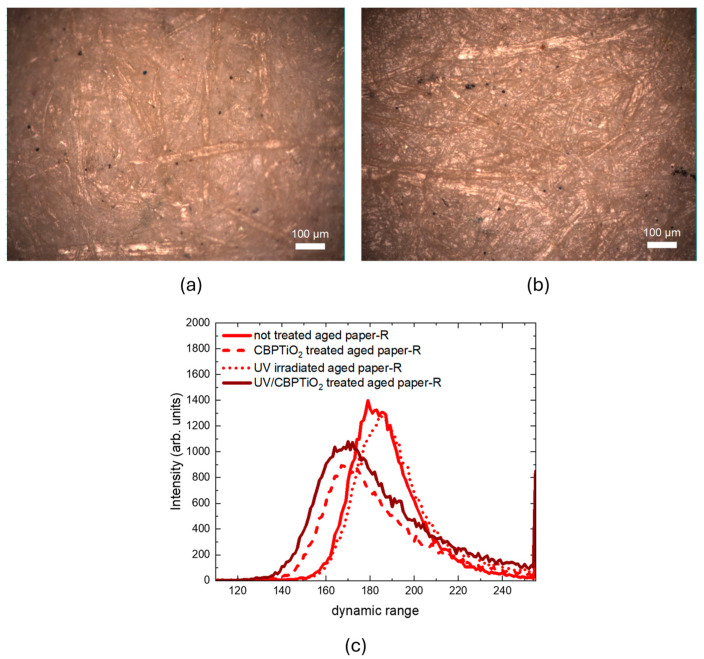
Optical images of 2021 paper naturally aged in laboratory: (**a**) uncoated; (**b**) coated with a thin layer of CBP/TiO_2_. (**c**) Red value distributions of 2021 paper naturally aged in laboratory: uncoated paper (solid line), CBP/TiO_2_ coated paper (dashed line), uncoated UVA-irradiated paper (dotted line), CBP/TiO_2_ coated UVA-irradiated paper.

The same procedure was performed on the 2021 paper that had undergone the natural aging processes in the laboratory environment (see Figure 11a,b). In this case, as shown in Figure 11c for the red values of RGB distributions, the clearing effect of CBP/TiO_2_ was preserved when exposed to UV radiation. The green and the blue distributions follow the same behavior of red values.

**Figure 12:**
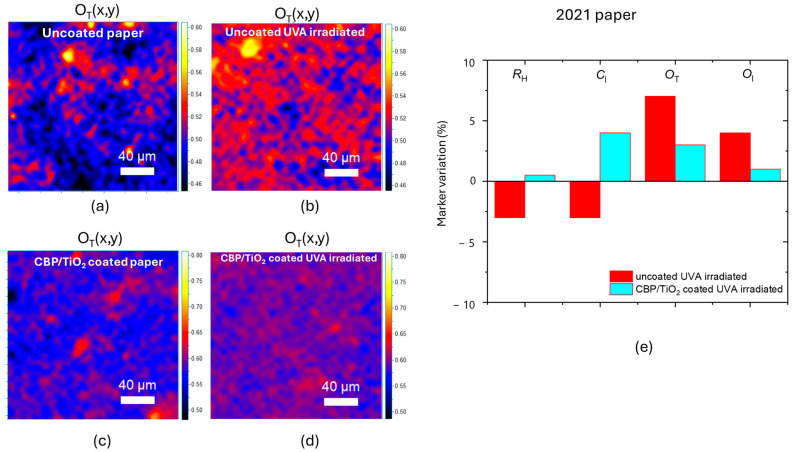
O_T_ marker value maps for (**a**) uncoated paper, (**b**) UVA-irradiated uncoated paper, (**c**) coated paper, (**d**) UVA-irradiated coated paper. (**e**) Marker variation in percentage after 31 h of UVA irradiation for the uncoated and CBP/TiO_2_ coated 2021 paper exposed to ambient light of laboratory for three years.

The *O*_T_ marker variation, shown in Figure 12a–d, highlights minimal degradation for samples coated with the CBP/TiO_2_ composite. Specifically, the CBP/TiO_2_ coating induces only a 3% increase in the *O*_T_ marker following UVA irradiation (Figure 12e). In comparison, the uncoated 2021 paper exhibits a 7% increase in the *O*_T_ marker, which is slightly lower than the value observed for modern paper. The difference can be explained by the fact that the 2021 paper sample had already been exposed to ambient light for three years, in which the oxidation process progressed rapidly during its initial stage and later slowed down, as is typical. It is worth noticing that in this case, the application of CBP/TiO_2_ coating produces an increase in *R*_H_ and *C*_I_ markers; this can be due to the formation of hydrogen bonds between fibers as previously discussed.

Overall, the effect of CBP/TiO_2_ coating on 2021 paper is to reduce the light induced oxidation and counteract the hydrolysis process.

**Figure 13:**
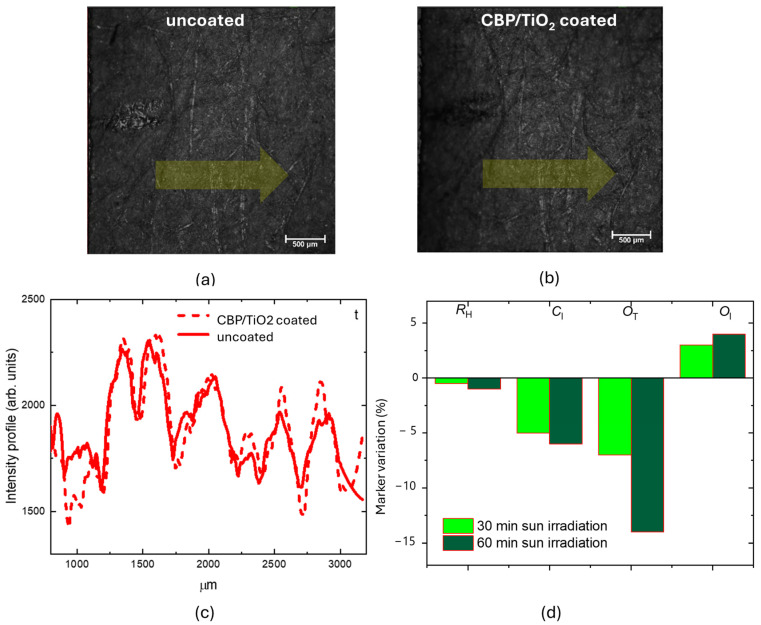
CLSM images obtained in reflection mode of the 2021 paper: (**a**) uncoated; (**b**) CBP/TiO_2_-coated; (**c**) the intensity profiles measured along the yellow arrow; (**d**) marker variation percentage after 30 and 60 min of outdoor sun irradiation.

To further assess the protective properties of the composite gel, an additional 2021 paper sample was prepared and coated with a thicker layer of CBP/TiO_2_. Figure 13 shows CLSM images, in reflection mode, comparing the same region of uncoated (Figure 13a) and CBP/TiO_2_ coated (Figure 13b) paper samples under identical acquisition conditions. A short pencil line, visible on the left side of the images, was used as spatial reference to facilitate microscope observation of the same area before and after treatment.

The slightly increased thickness of the coating layer does not alter the optical image resolution of the paper, as confirmed by the intensity profiles measured along the yellow arrow shown in Figure 13c. The samples were exposed to outdoor sunlight (22 W/m^2^, 16 May 2022) for 30 min and 1 h. A slight but progressive decrease in the *R*_H_ index was observed, due to hydrolysis processes. The *O*_T_ marker showed a progressive decrease with increased irradiation time. On the other hand, the *O*_I_ marker exhibited an increase, suggesting that although the photoactivated TiO_2_ decomposes organic oxidizing acids, the oxidation process continues, forming additional C=O bonds [7].

## 3. Discussion

The aim of the present study was to evaluate the effectiveness of cleaning and protective treatments using a CBP/TiO_2_ composite gel activated by UV light, through a Raman spectroscopy-based diagnostic protocol.

Regarding the cleaning action, compared to the application of CBP alone, the addition of TiO_2_ slightly improves the removal of oxidizing functional groups, while also ensuring biocidal activity. Moreover, by using CLS fitting of Raman spectra, we were able to monitor the effectiveness of gel manual removal.

To assess the ability of the CBP/TiO_2_ coating to shield paper surfaces from light aging, we exposed various paper samples, coated and uncoated, to UVA radiation.

Our measurements show that, in the coated 2024 paper samples, hydrolysis is slowed down, and oxidizing agents are progressively removed through a self-cleaning process, while the protective layer is gradually depleted during exposition. In the case of 2021 paper naturally aged in the laboratory, the CBP/TiO_2_ coating acts as paper consolidant (increase in *R*_H_ and *C*_I_ marker) counter acting the light induced oxidation. Applying a thicker layer of composite coating on 2021 paper, the same self-cleaning process observed for the 2024 modern paper occurred, even when the 2021 paper was exposed to outdoor sunlight.

Additionally, in all the experiments, the presence of the coating layer does not alter the original color of the paper and prevents light-induced yellowing, maintaining RGB values of paper.

## 4. Materials and Methods

### 4.1. Nanocomposite Hydrogel Preparation

Carbopol^®^ Ultrez 10 (CAS No. 9003-01-4) from Lubrizol (Wickliffe, OH, USA) has a high level of purity. The following specifications were reported by the manufacturer: loss on drying ≤ 2%, heavy metals ≤ 10 ppm, and residual solvents ≤ 0.45%.

A 3.33 wt% solution of Carbopol^®^ in water was used for the hydrogel matrix. Since Carbopol^®^ reaches maximum viscosity at pH 7, a 20 wt% NaOH solution was employed as a neutralizing agent [25,26,27]. To achieve a homogeneous texture, TiO_2_ nanoparticles were incorporated into the mixture, followed by sonication to ensure uniform dispersion. The resulting gel proves to be suitable for easy application on substrates. Furthermore, it holds long-term stability and retains its properties even at elevated temperatures, provided it is stored in a sealed container shielded from light.

**Figure 14:**
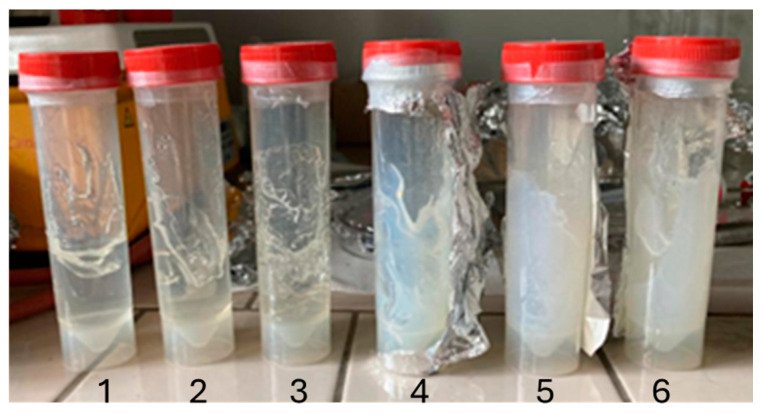
Photo of hydrogel. On the left CBP gel (1–2–3) on the right CBP/TiO_2_ gel (4–5–6).

The primary preparation conditions for the composite hydrogel are summarized in Table 1 and the resulting hydrogel structure is illustrated in Figure 14.

Titania nanoparticles employed were synthesized via CO_2_ laser-induced pyrolysis reactions in a titanium(IV) isopropoxide aerosol, following a previously established protocol [20]. This method ensures high chemical purity of the nanoparticles, as the reactor walls remain cold and non-reactive throughout the process. An estimation carried out through BET measurements shows a mean powder diameter of 20 nm.

When irradiated with photons of energy greater than its band gap, TiO_2_ undergoes excitation and generates free electron–hole pairs:

(5) $${\mathrm{TiO}}_{2}+h\nu \to {\mathrm{TiO}}_{2-}$$

These electron–hole pairs persist briefly before recombination, leaving the molecule in an excited state long enough to allow for them to interact with chemical species adsorbed on the NP surface. Organic contaminants are therefore removed by redox reactions [17,28].

### 4.2. Paper Samples

Paper samples of 100 cm^2^ were cut from 2024 and 2021 laser printing paper. The 2021 sample was exposed for three years to ambient light. Additionally, we examined non printed areas of an ancient book (Brehm, Vita degli Animali, vol. 6, Torino 1896). The book was positioned on the sample holder.

### 4.3. Gel Application Procedure

The samples were divided into two subsamples and treated with CBP and CB10_TiO_2_ composite gel. The gels were applied as a homogeneous layer using a spatula. In the case of the CB_10__TiO_2_ composite gel, the sample was then placed under a UV light source (LED lamp) to activate the TiO_2_ nanoparticles. After treatment, the gel was easily removed with the use of a spatula. Contact times for both cleaning procedures were set at 15 min and 1 h.

### 4.4. UV Source

The photocatalytic process of titania nanoparticles was activated with a high power UV LED source emitting a peak wavelength at 385 nm. The LED was used by mounting a collimation optics obtaining a closely collimated beam with a power of 120 mW on a beam area of 1450 mm^2^. This UV LED was used also for light ageing process.

### 4.5. Raman Confocal Spectrometer

Raman spectra were acquired using a confocal micro-Raman spectrometer (XploRA Plus, Horiba, Lille, France SAS) with a 532 nm laser wavelength. The Raman signals were collected through a microscope equipped with 5×, 10×, 50×, and 100× objectives. Laser power was adjusted using neutral density filters. After preliminary studies, optimal conditions for both laser power and acquisition times were selected to achieve good signal-to-noise ratio while ensuring safe operating conditions for the samples. Raman spectra were recorded point-by-point across a selected area, following a predefined grid defining a 2D map and defining a 2D spectra array.

The fluorescence background was automatically subtracted from each Raman spectrum, for which the software also calculated the intensity, peak area, and peak width, which were used as contrast parameters. In the case of partially overlapping bands, the software performed spectral deconvolution, obtaining the spectrum as the best fit of a superposition of Gaussian curves. For each defined contrast parameter, a 2D Raman map was generated and associated with the optical image collected.

### 4.6. Confocal Laser Scanning Optical Microscope

Optical images were acquired using a confocal laser scanning microscope (CLSM) Nikon 80i-C1 (Amstelveen, The Netherlands), operating in both fluorescence and reflection modes [29]. In fluorescence mode, the samples were illuminated with a continuous 445 nm laser (nominal output power of 1.5 mW), and the spectrally integrated PL signal was detected by a system of two photomultiplier tubes, which separately and independently acquired signals in two distinct visible spectral ranges (red and green), selected using an optical filtering system: a long-pass filter at 560 nm for the red signal and a filter transmitting from 500 nm to 530 nm for the green signal. In reflection mode, the samples were illuminated using a 532 nm laser (nominal output power of 3 mW), and the reflected signal was detected by a PMT.

### 4.7. Analysis with ImageJ Software of Optical Microscope Images

The ImageJ processing used for RGB analysis of the optical images is based on the histogram elaboration for the separated color channels R, G, and B providing their value distributions. An identical region of interest was selected for all the samples in order to be able to correctly evaluate the results, with the same conditions, minimizing the effects of possible systematic non-uniformity of illumination.

## 5. Conclusions

Ancient books, artworks, and important documents preserved in archives are subject to chemical and biological deterioration processes, which can lead to irreversible damage.

Several hydrogel cleaning treatments are being studied to slow down these processes without altering the properties of the paper, while the existing literature suggests the potential of titanium dioxide nanoparticles for protecting artworks from light-induced degradation. In this study, we synthesized CBP/TiO_2_ composite hydrogel and evaluated its effectiveness as cleaning and protective agent for paper artworks, employing a non-invasive diagnostic protocol based on Raman spectroscopy. Prolonged exposure to UVA radiation was used to simulate ageing and/or damaging effects.

It has been observed that papers coated with the composite gel layer exhibit better color stability, compared to uncoated ones, when exposed to UVA radiation; a lower content of oxidizing agents was observed as well as inhibition or elimination of hydrolysis process. Moreover, the application of CBP/TiO_2_ coating does not alter the RGB values of the paper making this treatment of potential interest for cultural paper artifacts.

## Figures and Tables

**Table 1:** Materials for hydrogel preparation.

Material	Structure	Function	Concentration or Volume
Carbopol^®^ Ultrez 10	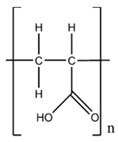	Polymer	3.33 wt%
Deionized water	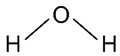	Solvent	15 mL
Sodium hydroxide	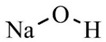	Neutralizing agent	260 μL
Titanium dioxide	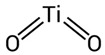	Photoactive element	800 ppm

## Data Availability

All data are available from the corresponding authors upon reasonable request.
